# Tocilizumab Treatment for Microvascular Inflammation and Chronic Active Antibody-mediated Rejection in Kidney Transplantation

**DOI:** 10.1097/TXD.0000000000001867

**Published:** 2025-10-03

**Authors:** Edmund Huang, Michie Adjei, Alice Peng, Reiad Najjar, Jun Shoji, Sindhu Chandran, Ashley Vo, Stanley C. Jordan

**Affiliations:** 1 Division of Nephrology, Department of Medicine, Comprehensive Transplant Center, Cedars-Sinai Medical Center, West Hollywood, CA.; 2 Department of Surgery, Comprehensive Transplant Center, Cedars-Sinai Medical Center, West Hollywood, CA.

## Abstract

**Background.:**

Interleukin-6 (IL-6) is an important driver of humoral immunity and a target for treatment of antibody-mediated rejection (AMR) in kidney transplantation. Data on IL-6 inhibition for treatment of AMR are mixed and their efficacy remains inconclusive. In this retrospective observational study, we investigated the association of monthly tocilizumab infusions with the trajectory of estimated glomerular filtration rate (eGFR) among kidney transplant patients with histologic features of chronic active AMR.

**Methods.:**

Through institutional database review, 85 adult kidney transplant patients with histologic features of chronic active AMR treated with monthly tocilizumab 8 mg/kg intravenous infusions were identified. Piecewise linear mixed effects models were fitted to compare eGFR trajectories 12 mo before and after tocilizumab initiation.

**Results.:**

The eGFR declined at a rate of –0.70 mL/min/1.73 m^2^/mo (95% confidence interval, –1.03 to –0.36) preceding tocilizumab initiation and stabilized after treatment onset to a slope of –0.07 (–0.35 to 0.21) mL/min/1.73 m^2^/mo (slope difference: 0.59 [0.22-0.97] mL/min/1.73 m^2^/mo, *P* = 0.002).The distribution of the sum mean fluorescence intensity of donor specific antibodies (DSA) among 65 DSA^+^ patients remained unchanged from baseline to 1 y after treatment; however, 14 of 65 DSA^+^ patients (22%) no longer had DSA at 1 y. There was 1 graft loss and 2 deaths, both COVID-19-related, by 12 mo after treatment onset.

**Conclusions.:**

This study suggests that monthly treatment with the anti-IL-6 receptor monoclonal antibody, tocilizumab, may stabilize allograft function among kidney transplant patients with chronic active AMR and that further studies to confirm its efficacy should be conducted.

## INTRODUCTION

Interleukin-6 (IL-6) is a mediator of inflammation and plays an important role in humoral responses and autoimmune diseases. IL-6 stimulates T follicular helper cells, driving maturation of B cells to plasmablasts and long-lived antibody-producing plasma cells. In kidney transplantation, IL-6 production is strongly associated with the presence of donor-specific antibodies (DSAs), linking the IL-6 pathway to antibody-mediated rejection (AMR).^1^ Binding of DSA to HLA targets on graft endothelium initiates IL-6 production that stimulates intimal proliferation and obliterative vasculopathy, which are often seen in chronic AMR.^2^ In addition, IL-6 is important for class switch from IgM to IgG after immune activation. Given these observations, there is considerable interest in targeting the IL-6 pathway for treatment of AMR in kidney transplantation.

Tocilizumab is an IL-6 receptor antagonist approved by the United States Food and Drug Administration for the treatment of rheumatoid arthritis, polyarticular juvenile idiopathic arthritis, giant cell arteritis, and chimeric antigen receptor T cell–induced cytokine release syndrome. Studies using tocilizumab off-label for desensitization and treatment of chronic active AMR in kidney transplantation were promising and served as the basis for a large double-blinded, placebo-controlled randomized clinical trial investigating the efficacy of clazakizumab, a monoclonal antibody directed against soluble IL-6, for treatment of chronic active AMR (IMAGINE study, NCT03744910).^3^ Interim analysis of 1 y data indicated that the primary endpoint of time to composite all-cause graft loss or irreversible loss of allograft function would not be met, leading to early termination of the trial for futility.^4^ These results contrasted with earlier trial data of clazakizumab for treatment of chronic active AMR, which observed stabilization of estimated glomerular filtration rate (eGFR), reduction in DSA strength, and improvement in the molecular AMR profile.^5,6^ Therefore, at present, it remains inconclusive whether IL-6 inhibition is an effective strategy for the treatment of chronic active AMR.

We have used tocilizumab at our institution for the treatment of kidney transplant patients in varying contexts, including pretransplant desensitization, prevention of posttransplant DSA rebound, treatment of DSA with and without AMR, and treatment of microvascular inflammation (MVI) with and without DSAs. Given the recent IMAGINE study results and the equipoise concerning the efficacy of IL-6 blockade as a relevant target in kidney transplantation, we undertook this study to investigate the effect of tocilizumab treatment on allograft function in kidney transplant patients with chronic active AMR.

## MATERIALS AND METHODS

This article adheres to the Declaration of Istanbul, and all donor kidneys involved in this study conformed to standard transplant allocation practices in the United States. The study was approved by the Cedars-Sinai Medical Center Institutional Review Board and was not subject to the requirements for informed consent (Pro00048317).

### Study Population

All adult patients (age 18 y or older) with histologic features of chronic active AMR defined by Banff 2022 criteria and treated with tocilizumab between 2013 and 2023 were identified. Histologic criteria included (1) morphologic evidence of chronic tissue injury consisting of transplant glomerulopathy (Banff cg>0) or severe peritubular capillary basement membrane multilayering and (2) evidence of current/recent antibody interaction with the vascular endothelium, including peritubular capillary linear C4d staining (C4d≥2 by IF on frozen sections) or at least moderate MVI (g+ptc≥2 with at least g≥1 in the presence of concomitant acute or borderline T cell–mediated rejection [TCMR]).^7^ Patients were categorized into 1 of 2 groups: AMR^+^ (meeting Banff 2022 histologic criteria for chronic active AMR with concurrent DSA and/or C4d^+^) and MVI^+^/AMR^–^ (MVI without DSA). Patients diagnosed with chronic active AMR within 90 d of transplant were excluded.

### Study Exposure

After meeting inclusion criteria, including no history of diverticulitis and/or inflammatory bowel disease, all patients were prescribed tocilizumab 8 mg/kg intravenously monthly and followed up to 12 mo from the start of treatment. Early in our experience, we prescribed monthly tocilizumab in 6-mo intervals, but our practice evolved over time to indefinite monthly treatment in the absence of side effects or adverse events. Infusions were administered for 30 min through a peripheral intravenous line without premedications. Per center practice, we did not give antimicrobial prophylaxis against *Pneumocystis jirovecii* or cytomegalovirus. A complete blood count and liver function tests were monitored 2 wk before and 2 wk after each infusion. Tocilizumab infusions were withheld for a white blood cell count <2000/UL, platelets <100 000/UL, or aspartate aminotransferase/alanine transaminase ≥3 times the upper limit of normal.

### Outcome Measure

The primary outcome was the change in eGFR slope during the 12 mo before and after tocilizumab initiation. eGFR was derived from creatinine values in the electronic medical record and calculated using the Chronic Kidney Disease Epidemiology Collaboration race-free creatinine equation (2021).^8^ Secondary outcomes included a comparison of eGFR slope before and after tocilizumab initiation in both the AMR^+^ and MVI^+^/AMR^–^ groups, those with and without DSA at treatment onset, and among patients with mild versus advanced interstitial fibrosis and tubular atrophy (IF/TA) on biopsy. Additional secondary outcomes included patient and allograft survival within 1 y of treatment initiation, change in DSA mean fluorescence intensity (MFI) before and after treatment with tocilizumab, and the rate of infections during tocilizumab treatment.

### Statistical Analysis

Baseline characteristics are expressed as proportions for categorical data and medians (interquartile ranges, IQRs) for continuous data and are presented for the overall population and separately for the AMR^+^ and MVI^+^/AMR^–^ groups.

Piecewise linear mixed effects models using random slopes and intercepts with a knot at time zero representing the day of tocilizumab initiation were constructed to compare the eGFR trajectory and slope before and after tocilizumab initiation. All eGFRs recorded within 12 mo before and after tocilizumab initiation were included in the analysis. For patients who initiated tocilizumab within the first posttransplant year, eGFRs measured before 90 d posttransplant were excluded to exclude eGFR changes related to postoperative recovery of kidney function in the model. Subgroup analyses were performed to assess the eGFR trajectory separately for the AMR+ and MVI^+^/AMR^–^ groups, for those with and without DSA at treatment initiation, and according to histologic Banff scores.

All *P* values were 2-tailed and a *P* value of <0.05 was considered significant. Statistical analyses were conducted using Stata version 14.2 (College Station, TX).

## RESULTS

### Baseline Characteristics

**FIGURE 1.**
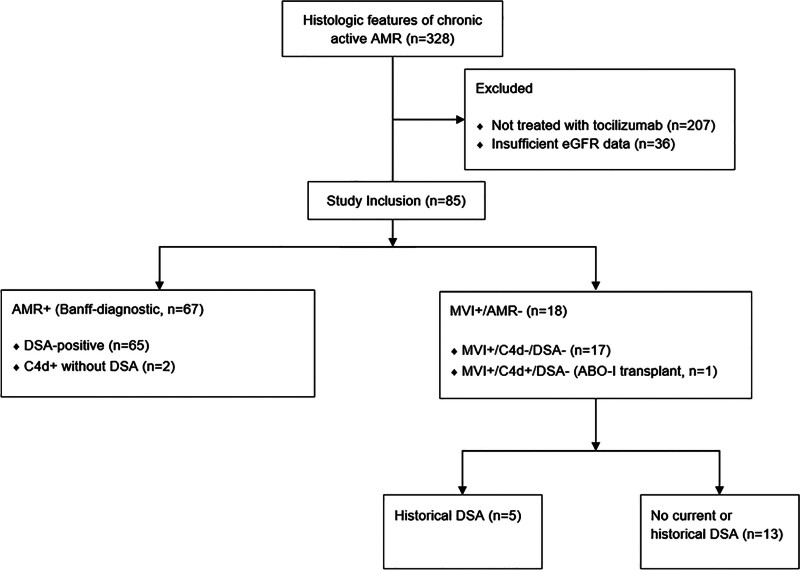
Study flowchart. AMR, antibody-mediated rejection; DSA, donor-specific antibody; eGFR, estimated glomerular filtration rate; MVI, microvascular inflammation.

Figure 1 shows the study flowchart. Between February 14, 2013, and May 1, 2023, a total of 85 patients with histologic features of chronic active AMR and treated with tocilizumab were identified. Of these, 67 met the Banff 2022 criteria for chronic active AMR (AMR^+^ group; DSA^+^, n = 65; C4d^+^ without DSA, n = 2) and 18 were included in the MVI^+^/AMR^–^ group who did not meet the diagnostic criteria for active AMR. Thirty-eight of 65 DSA+ (58%) patients had de novo DSA detected within 12 mo before tocilizumab initiation. All cases in the MVI^+^/AMR^–^ group were MVI^+^/C4d^–^/DSA^–^ except for 1 blood type B recipient of a blood type A2 donor who had C4d^+^ on biopsy. In this case, the C4d^+^ was not considered as meeting criteria for AMR given the length of time posttransplant (>3 y), which is well beyond the time when blood group incompatibility is relevant for AMR. Among the MVI^+^/AMR^–^ group were 5 patients who had historical DSA but were DSA^–^ at the time of diagnosis, thus not meeting the Banff criteria for active AMR but meeting the criteria for chronic AMR.

Baseline characteristics of the overall study population and for the AMR^+^ and MVI^+^/AMR^–^ group are shown in Table 1. Patients received a median of 6 doses of tocilizumab (IQR, 5–8 doses) for a median of 349 (IQR, 324–361) d of follow-up. The median time posttransplant at treatment initiation was 6.6 (2.9–10.7) y and was not different between the AMR^+^ and MVI^+^/AMR^–^ groups. Seventy (82%) patients discontinued therapy within 12 mo of treatment initiation. The most common reasons for discontinuation were completion of the prescribed treatment course (44/70; 63%), infection-related concerns (7/70; 10%), treatment nonresponsiveness (6/70; 9%), and patient choice/change in insurance coverage (5/70; 7%). Only 1 patient discontinued treatment because of a noninfection-related adverse effect (transaminitis).

**TABLE 1.** Baseline characteristics

Characteristics	Overall (N = 85)	AMR^+^ (N = 67)	MVI^+^/AMR^–^ (N = 18)
Age, y, median (IQR)	49 (37–59)	49 (37–59)	52 (36–61)
Sex, male, n (%)	46 (54)	37 (55)	9 (50)
Race/ethnicity			
White	29 (34)	22 (33)	7 (39)
Black	15 (18)	12 (18)	3 (17)
Hispanic	35 (42)	29 (43)	6 (33)
Asian	5 (6)	4 (6)	1 (6)
Other	1 (1)	0 (0)	1 (6)
Concomitant immunosuppression, n (%)			
Tacrolimus	76 (89)	58 (87)	18 (100)
Cyclosporine	6 (7)	6 (9)	0 (0)
Mycophenolate	78 (92)	63 (94)	15 (83)
Prednisone	84 (99)	66 (99)	18 (100)
Belatacept	1 (1)	1 (1)	0 (0)
Sirolimus	4 (5)	3 (4)	1 (6)
Time posttransplant at treatment start, y, median (IQR)	6.6 (2.9–10.7)	6.6 (2.9–11.0)	6.3 (2.9–9.9)
eGFR at treatment start, mL/min/1.73 m^2^, median (IQR)	47 (38–62)	49 (37–64)	45 (40–52)
<30	11 (13)	8 (12)	3 (17)
30–59	49 (58)	38 (57)	11 (61)
≥60	25 (29)	21 (31)	4 (22)
DSA, n (%)	65 (76)	65 (97)	–
None	20 (24)	2 (3)	18 (100)
Preexisting only	24 (37)	24 (36)	–
De novo only	38 (58)	38 (57)	–
Both preexisting and de novo	3 (5)	3 (4)	–
Sum DSA MFI, median (IQR)	–	10 000 (5000–17 500)	–
Class I only	–	9 (14)	–
Class II only	–	46 (71)	–
Both class I and class II	–	10 (15)	–
Banff scores^*a*^			
g>2	25 (49)	16 (44)	9 (60)
ptc>2	45 (88)	32 (89)	13 (87)
C4d positive	17 (33)	16 (44)	1 (7)^*b*^
ci>2	15 (29)	11 (31)	4 (27)
ct>2	15 (29)	11 (31)	4 (27)

^*a*^Banff scoring only available on 51 patients (AMR^+^: 36; MVI^+^/AMR^–^: 15).

^*b*^ABO-incompatible transplant.

AMR, antibody-mediated rejection; DSA, donor-specific antibody; eGFR, estimated glomerular filtration rate; IQR, interquartile range; MFI, mean fluorescence intensity.

Banff scores were recorded in our institutional database beginning in 2017 and were available for 51 patients (AMR^+^: 36; MVI^+^/AMR: 15). Most patients had at least moderate peritubular capillaritis (ptc≥2; 88%), with fewer having moderate or severe glomerulitis (g≥2; 49%). Advanced IF (ci≥2) and TA (ct≥2) were present in only 29% of cases.

Approximately half of the patients (47/85; 55%) received IVIG in proximity to tocilizumab, within 3 mo of treatment initiation. Of these, 36 of 47 (77%) had received more remote AMR treatments with IVIG and rituximab (30/47; 64%) in keeping with our typical usage of tocilizumab as a rescue treatment for patients not responsive to previous treatments. Among patients who did not receive IVIG within 3 mo of tocilizumab, 29 of 38 (76%) had received prior IVIG and 30 of 38 (79%) had been treated with prior rituximab. Additional prior treatments for AMR included plasmapheresis (29/85; 34%) and eculizumab (5/85; 6%).

### Comparison of eGFR Trend Before and After Tocilizumab Treatment Initiation

**FIGURE 2.**
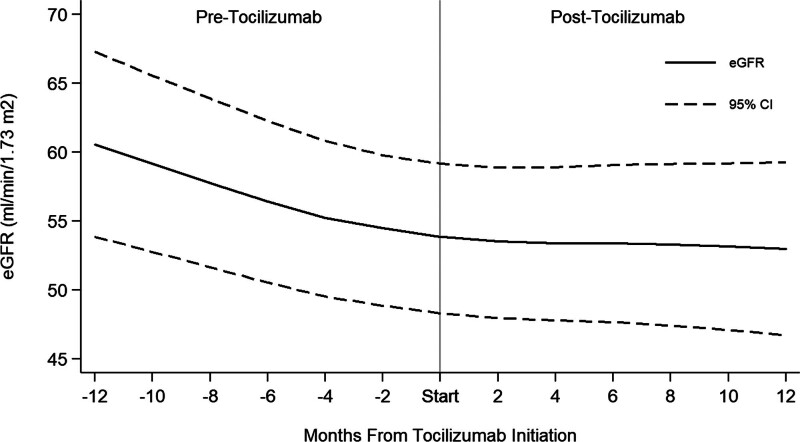
Trend in eGFR (mL/min/1.73 m^2^) with 95% CIs before and after tocilizumab initiation. CI, confidence interval; eGFR, estimated glomerular filtration rate.

In the overall study population, the median eGFR was 47 (38–62) mL/min/1.73 m^2^ at treatment initiation. Table 2 presents a comparison of eGFR slopes during 12 mo before and after tocilizumab initiation. Using a linear mixed-effects model, kidney allograft function declined during the 12 mo before tocilizumab initiation with a negative pretreatment eGFR slope (–0.70 [–1.03 to –0.36] mL/min/1.73 m^2^/mo). Tocilizumab initiation coincided with stabilization of kidney allograft function during the ensuing 12 mo. The eGFR slope post–tocilizumab initiation was –0.07 (–0.35 to 0.21) mL/min/1.73 m^2^/mo, which was significantly different than the pretreatment eGFR slope (slope difference, 0.59 [0.22–0.97] mL/min/1.73 m^2^/mo, *P* = 0.002). A graphical representation of the model-based eGFR trajectory before and after tocilizumab initiation is depicted in Figure 2.

**TABLE 2.** Comparison of eGFR slope before and after tocilizumab treatment initiation

Model	Slope eGFR, mL/min/1.73 m^2^/mo	95% Confidence interval	*P* (before vs after)
Overall population			0.002
Before	–0.70	–1.03 to –0.36	
After	–0.07	–0.35 to 0.21	
AMR^+^			0.02
Before	–0.70	–1.05 to –0.34	
After	–0.17	–0.45 to 0.12	
MVI^+^/AMR^–^			0.06
Before	–0.66	-1.45 to 0.13	
After	0.32	–0.62 to 1.26	
DSA^+^			0.02
Before	–0.74	–1.12 to –0.36	
After	–0.16	–0.43 to 0.10	
DSA^–^			0.06
Before	–0.58	–1.25 to 0.09	
After	0.23	–0.56 to 1.03	
eGFR <30 mL/min/1.73 m^2^			<0.001
Before	–1.90	–3.10 to –0.71	
After	0.16	–0.21 to 0.52	
eGFR 30–59 mL/min/1.73 m^2^			0.04
Before	–0.76	–1.03 to –0.49	
After	–0.26	–0.65 to 0.12	
eGFR ≥60 mL/min/1.73 m^2^			0.93
Before	0.07	–0.65 to 0.79	
After	0.14	–0.40 to 0.68	
ci/ct <2			0.20
Before	–0.32	–0.86 to 0.21	
After	0.13	–0.43 to 0.68	
ci/ct ≥2			0.008
Before	–1.75	–2.66 to –0.84	
After	–0.46	–1.02 to 0.09	

AMR, antibody-mediated rejection; DSA, donor-specific antibody; eGFR, estimated glomerular filtration rate; MVI, microvascular inflammation.

Subgroup analyses of eGFR trends before and after tocilizumab treatment are shown in Table 2. The eGFR trends pre- and posttreatment were similar to those of the overall population in the AMR^+^ and MVI^+^/AMR^–^ groups and among patients with and without DSAs at the time of tocilizumab initiation, where a decline in eGFR was observed before tocilizumab initiation, with subsequent flattening of the eGFR slope after treatment onset. This observation was also seen when the analysis was stratified by the degree of IF/TA on biopsy, although a statistically significant difference in eGFR slope was only observed among cases with more advanced IF/TA (ci/ct ≥2) and not in cases with milder IF/TA (cict<2). A treatment effect was observed among patients with eGFR <60 mL/min/1.73 m^2^ but not among those with eGFR ≥60 mL/min/1.73 m^2^, although these observations should be interpreted with caution given relatively smaller numbers of patients within these subgroups.

### Graft and Patient Survival

Only 1 graft failure developed during the study period in an AMR^+^ patient, 362 d after tocilizumab initiation. The cause of graft failure was deemed related to progressive rejection. There were 2 deaths during the study period, both related to COVID-19 infections.

### Evolution of DSAs

The median number of DSAs among the 65 patients with DSA at treatment onset was 1 (range, 1–7; IQR, 1–2), and the median sum MFI of DSAs per patient was 10 000 (range, 2500–71 250; IQR, 5000–17 500). At 1 y after treatment initiation, statistically fewer DSAs per patient were observed compared with those at treatment onset (median, 1; range, 0–5; IQR, 1–2; *P* < 0.001).

**FIGURE 3.**
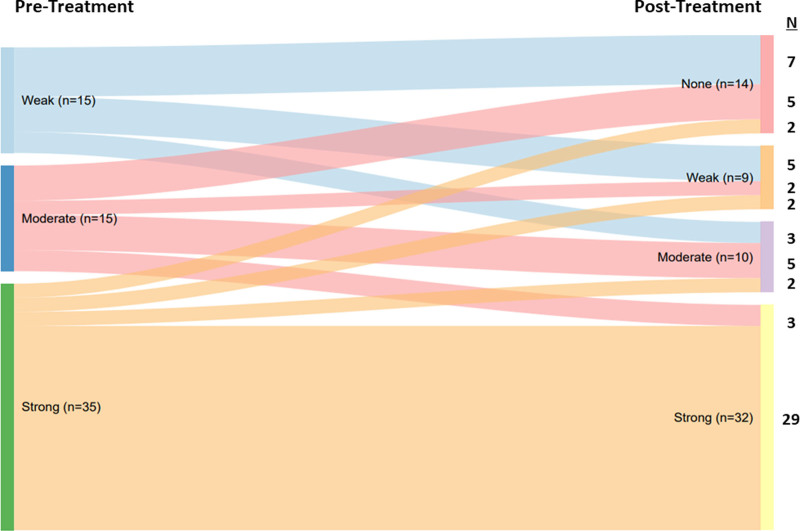
DSA sum MFI strength categories before and after tocilizumab treatment (n = 65 patients with DSAs at treatment onset). Sum DSA MFI categories were defined as: weak: <5000, moderate: 5000 to <10 000, and strong: ≥10 000. DSA, donor-specific antibody; MFI, mean fluorescence intensity.

Among 65 patients with DSAs at treatment onset, the sum DSA MFI at 1 y (median, 8750; range, 0–78 750; IQR, 2500–17 500) was not different compared with the baseline sum DSA MFI (median, 10 000; range, 2500–71 250; IQR, 5000–17 500; *P* = 0.10). Nevertheless, 14 of 65 DSA^+^ patients (22%) no longer had detectable DSA post–tocilizumab treatment. Of these, 7 had weak (MFI <5000), 5 had moderate (MFI 5000 to <10 000), and 2 had strong DSA (MFI ≥10 000) strength at treatment onset. Figure 3 shows the distribution of DSA strength by MFI strength category before and 12 mo after tocilizumab initiation. Post–tocilizumab treatment, fewer patients had moderate and strong DSA, although the difference was not statistically significant.

One patient developed a de novo moderate strength (MFI 5000) class I DSA by 1 y after treatment; 2 others had a rebound of historical DSAs that were not present at treatment onset.

### Infections During Tocilizumab Treatment

Infections experienced from the onset of tocilizumab treatment extending to 6 mo after the last tocilizumab exposure are presented in **Table S1 (SDC,** https://links.lww.com/TXD/A797). A total of 75 infections developed among 47 of 85 patients (55%) during 118 person-years of follow-up, yielding an incidence rate of 0.64 infections/person-year. Most infections were minor and treated in the outpatient setting. The most common infections were upper respiratory infections (19/75; 25%) and urinary tract infections (17/75; 23%). Only 1 case of cytomegalovirus viremia and no cases of BK viremia or nephropathy were observed. Seven patients (8%) discontinued tocilizumab treatments because of infection concerns.

## DISCUSSION

In this study of patients with histologic features of chronic active AMR and declining eGFR, we observed that tocilizumab was associated with stabilization of eGFR during the 12 mo after treatment initiation. This observation was similar for both those meeting Banff criteria for AMR and among DSA^–^ patients with MVI on histology. In addition, 14 of 65 DSA^+^ patients no longer had detectable DSA by the end of the study period. These findings support observations from earlier studies suggesting a role for IL-6 inhibition in the treatment of chronic active AMR.

IL-6 is a relevant target for the treatment of antibody-mediated disorders given its role in driving B-cell proliferation and the maturation of naive B cells into plasmablasts and long-lived antibody-producing plasma cells. Both B cells and plasma cells express IL-6 receptors, and experimental models have observed that IL-6 blockade reduces IgG and DSA production^9,10^ and promotes allograft survival.^11^ Furthermore, IL-6 has been implicated in the development of allograft vasculopathy, a lesion characteristic of chronic rejection, which can be mitigated with IL-6 inhibition in experimental models.^12^ Importantly, recent data in humans and animal models show that anti-IL-6 treatment inhibits CD8^+^ T-effector/memory cells and induces T-regulatory cell populations, which are critical for the prevention of DSA rebound and rejection and extending graft survival.^13,14^

The current study is the largest series of patients treated with tocilizumab to date, including nearly as many patients as analyzed in the IMAGINE clazakizumab study (NCT03744910). Our findings support prior observations from our group and others, which have suggested a salutary role for IL-6 antagonism in the treatment of AMR. We previously reported that tocilizumab was associated with prolonged kidney allograft survival among 36 patients offered tocilizumab as “rescue therapy” after unsuccessful treatment with IVIG and rituximab with or without plasmapheresis.^3^ This early experience was supported by later studies showing stabilization of eGFR,^15-17^ reduction in DSA MFI,^16-18^ and, in some cases, histologic improvement.^16^ Similar observations have been reported in both heart^19^ and lung transplantation.^20^

Nevertheless, the positive experiences with tocilizumab in the literature have not been universally observed, and some reports have described no effect of tocilizumab on eGFR,^18,21-23^ histology,^18,21-23^ or transcriptional profile.^22^ There may be several reasons for the variable experiences in the literature, including small study sample sizes, which limit their generalizability to the larger transplant population, absence of appropriate control groups, and heterogeneous inclusion criteria involving both DSA^+^ and DSA^–^ recipients in most studies.

Although we observed that tocilizumab treatment was associated with stabilization of eGFR in both DSA^+^ and DSA^–^ recipients in this study, the number of DSA^–^ patients is too small to make any conclusions on the efficacy of tocilizumab in the absence of DSA. Before 2017, MVI without DSA and C4d was considered suspicious for AMR by the Banff criteria and often attributed to non-HLA antibodies. However, more recent evidence suggests that non-HLA antibodies are not commonly associated with DSA^–^ MVI and implicates antibody-independent natural killer (NK) cell mechanisms instead as the cause of endothelial cell injury and MVI.^24,25^ Co-culture experiments of proximal tubular endothelial cells with allogeneic NK cells reveal increased IL-6 production,^26^ raising the possibility that anti-IL-6 therapy might block the downstream effects of NK-mediated injury. Further studies are necessary to assess the efficacy of tocilizumab in DSA^–^ MVI and to investigate whether other agents with activity against NK cells, such as anti-CD38 monoclonal antibodies,^27,28^ may be beneficial.

The IMAGINE study was a highly anticipated randomized controlled clinical trial investigating the efficacy of a soluble IL-6 antagonist, clazakizumab, compared with placebo for the treatment of chronic active AMR. This trial was terminated early for futility after an interim analysis assessing change in eGFR from baseline to week 52 deemed that the trial would likely not meet its primary efficacy endpoint of time to composite all-cause allograft loss or irreversible loss of allograft function. The trial report has not yet been published, and at present, it remains unclear why this study was unsuccessful, despite prior reports suggesting that clazakizumab would be efficacious.^5,6^ It is known that chronic active AMR is a heterogeneous condition with a variable course, rendering the selection of an appropriate control group problematic for clinical trials and potentially hindering the success of a randomized controlled trial. Graft survival prognoses associated with transplant glomerulopathy, the hallmark lesion of chronic AMR, vary by the combination of clinical, histologic, and immunologic characteristics, with the worst prognoses associated with lower eGFR at baseline, more proteinuria, presence of DSA, a higher degree of MVI, and more advanced chronic histologic lesions.^29^ Given the heterogeneity of chronic active AMR and the number of factors that impact its course, a clinical trial might not be appropriately balanced in unmeasured confounders if the sample size is inadequate. Although a randomized controlled trial is the gold standard for testing the efficacy of an intervention, a pre- and postintervention design, as used in this study, may have advantages, as it controls for the inherent variability between individual cases by measuring the within-individual effect of the intervention.

Although the IMAGINE trial has dampened enthusiasm for IL-6 inhibition in the treatment of chronic active AMR, it should be noted that the study used half the dose that was used in the 2 phase II trials of clazakizumab, both of which reported promising results on DSA strength and eGFR.^5,6^ The rationale for the reduced dose stemmed from infectious and diverticular complications seen in 1 of the trials, which led to a protocol amendment where the dose of clazakizumab was reduced to 12.5 mg in 2 patients with a history of diverticulosis.^5^ Because of concern for adverse complications at the 25 mg dose, the IMAGINE study used the 12.5 mg dose in all patients without preliminary data supporting its efficacy. Given that it is unclear whether greater efficacy would have been seen at the 25 mg dose, we feel that it is premature to conclude on the basis of the IMAGINE study that IL-6 antagonism is not a viable approach for the treatment of AMR and encourage further studies to be conducted.

Limitations of this study include the absence of a concurrent control group, which we believe is mitigated by the pre- and postintervention study design, as discussed previously. This was a retrospective study and is subject to selection bias, as the decision to treat with tocilizumab was made by physician’s discretion and not per protocol. Additionally, we did not assess longer-term effects on eGFR in this population, as the longer-term trajectory of eGFR is subject to many additional confounders and not directly comparable with the relatively short-term pretreatment eGFR trend. Finally, nearly half of the patients in this study were concomitantly treated with IVIG in proximity (within 3 mo) to tocilizumab initiation. Although we cannot rule out that IVIG treatment contributed to the stabilization of eGFR observed in this study, expert consensus is that no treatment has proven successful for the treatment of AMR.^30^ Notable is that although most patients in this study received prior treatments for AMR, including IVIG, the pre–tocilizumab eGFR decline of –0.69 mL/min/1.73 m^2^/mo was comparable with the –0.757 mL/min/1.73 m^2^/mo eGFR decrease observed in a multicenter modeling study of patients with AMR used for sample size calculations in the IMAGINE study.^31^ This suggests that the impact of further courses of IVIG would be minimal and argues that much of the treatment effect seen after tocilizumab initiation was probably related to IL-6 inhibition. Although this study does not prove that IL-6 inhibition changes the natural history of AMR, we believe that there is enough of a signal to warrant additional studies in kidney transplantation in light of the shortcomings of the IMAGINE study.

In summary, this study observed that tocilizumab treatment for patients with histologic changes of chronic active AMR and declining eGFR was associated with stabilization of eGFR after treatment initiation. This study supports a possible role for IL-6 inhibition in the treatment of chronic active AMR despite a recent negative randomized controlled clinical trial and indicates that further studies should be conducted.

## Supplementary Material


