# Supplementary material for: Tocilizumab Treatment for Microvascular Inflammation and Chronic Active Antibody-mediated Rejection in Kidney Transplantation
Source: Transplant Direct. 2025 Oct 3;11(11):e1867. doi: 10.1097/TXD.0000000000001867 (PMC12499727; doi:10.1097/TXD.0000000000001867)
Supplement: Supplementary file 1 [file txd-11-e1867-s001.pdf]

Supplemental Table S1. Summary of reported infections during tocilizumab treatment (observation period from date of tocilizumab initiation to six months following tocilizumab discontinuation, 118 person-years of follow-up).

**Infection site**	**Count**
Respiratory	36
Upper respiratory infection	19
COVID	12
Pneumonia	5
Urinary tract infection	17
Gastrointestinal	7
Bacteremia	5
Skin	5
Viral	3
Miscellaneous	2
Overall	75
